# Supplementary material for: EEG Correlates of Active Stopping and Preparation for Stopping in Chronic Tic Disorder
Source: Brain Sci. 2022 Jan 24;12(2):151. doi: 10.3390/brainsci12020151 (PMC8870153; doi:10.3390/brainsci12020151)
Supplement: Supplementary file 1 [file brainsci-12-00151-s001.zip › brainsci-1489725 supplementary.pdf]

Supplementary Figure S1

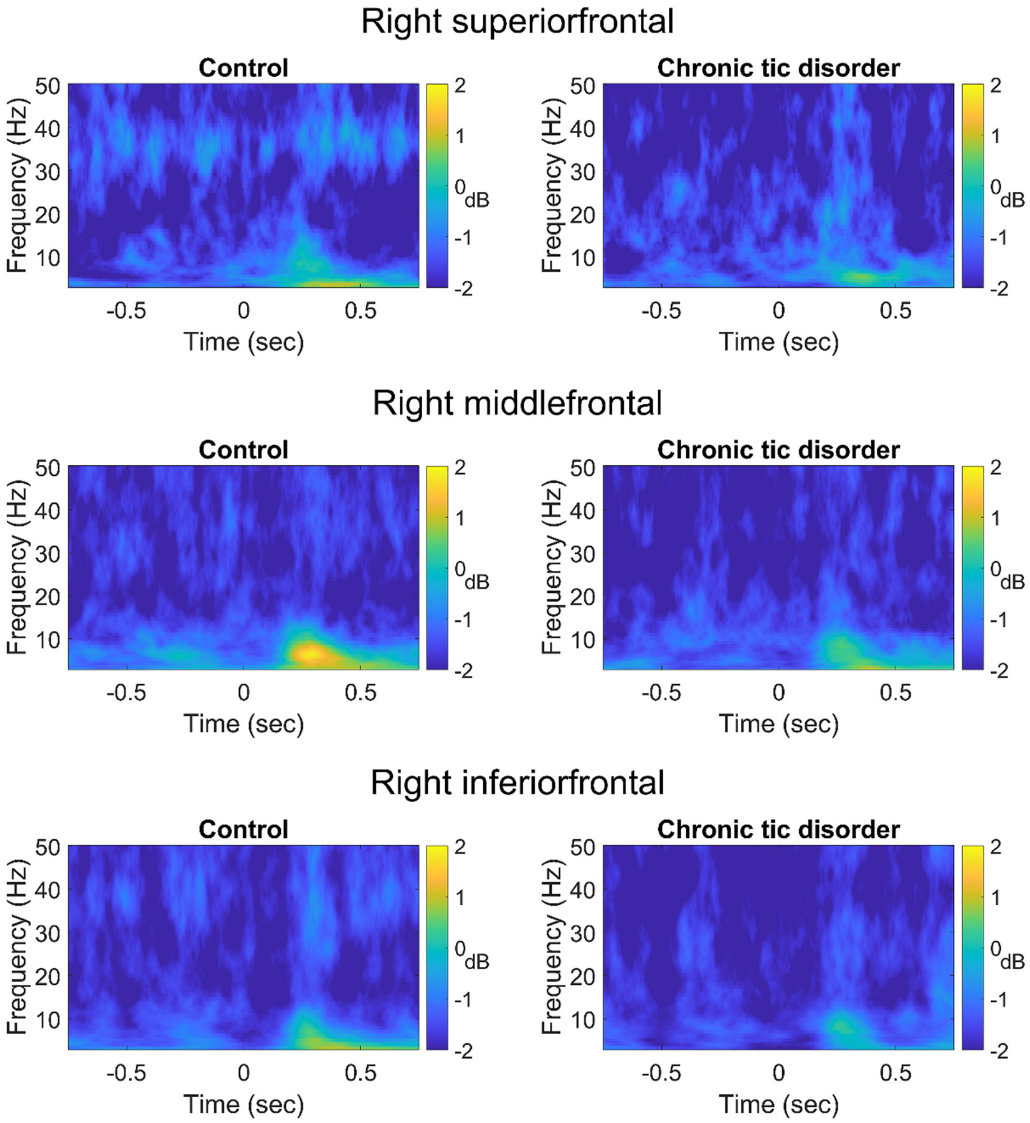

Successful stop trial event-related spectral perturbation images of frontal regions. Latency time 0 represents the stop cue. Both groups showed event related synchronization spanning all the analyzed frequencies in both right superiorfrontal, inferiormedial, and inferiorfrontal regions, with prominence in the θ and α frequency bands. No statistically significant difference was found between HC and CTD.
